# Dual Asymmetric Centrifugation Efficiently Produces a Poloxamer-Based Nanoemulsion Gel for Topical Delivery of Pirfenidone

**DOI:** 10.1208/s12249-020-01798-7

**Published:** 2020-10-02

**Authors:** Eugene P. Chung, Adrienne R. Wells, Mia Mae Kiamco, Kai P. Leung

**Affiliations:** Combat Wound Repair Division, United States Army Institute of Surgical Research, JBSA Fort Sam Houston, Texas 78234 San Antonio, USA

**Keywords:** poloxamer, dual asymmetric centrifugation, pirfenidone, antifibrotic, nanoemulsion

## Abstract

This study used dual asymmetric centrifugation (DAC) to produce a topical vehicle for Pirfenidone (Pf; 5-methyl-1-phenyl-2[1H]-pyridone)—a Food and Drug Administration-approved antifibrotic drug indicated for idiopathic fibrosis treatment. Pf was loaded (8 wt%) in a poloxamer nanoemulsion gel (PNG) formulation consisting of water (47.8 wt%), triacetin (27.6 wt%), poloxamer 407 (P407, 13.8 wt%), polysorbate 80 (1.8 wt%), and benzyl alcohol (0.9 wt%). To our knowledge, poloxamer gels are typically processed with either high-shear methods or temperature regulation and have not been emulsified using DAC. Using a single-step emulsification process, 2 min mixed at 2500 RPM resulted in the lowest Pf loading variability with a relative standard deviation (RSD) of 0.96% for a 1.5 g batch size. Batch sizes of 15 g and 100 g yield higher RSD of 4.18% and 3.05%, respectively, but still in compliance with USP guidelines. *Ex vivo* permeation in full thickness porcine skin after 24 h showed total Pf permeation of 404.90 ± 67.07 μg/cm^**2**^. Tested *in vitro* on human dermal fibroblasts stimulated with transforming growth factor-beta 1 (TGF-β1), Pf-PNG resulted in a > 2 fold decrease in α-SMA expression over vehicle control demonstrating that formulated Pf retained its biological activity. One-month stability testing at 25°C/60% relative humidity (RH) and 40°C/75% RH showed that % drug content, release kinetics, and biological activity were largely unchanged for both conditions; however, pH decreased from 6.7 to 5.5 (25°C/60% RH) and 4.5 (40°C/75% RH) after 1 month. Overall, these data demonstrate the utility of DAC to rapidly and reproducibly prepare lab-scale batches of emulsified gels for pharmaceutical formulation development.

**Electronic supplementary material:**

The online version of this article (10.1208/s12249-020-01798-7) contains supplementary material, which is available to authorized users.

## INTRODUCTION

Dual asymmetric centrifugation (DAC) is a bladeless mixing process that is efficient at blending viscous materials (*e.g.,* silicone adhesives) (1). Through a combination of centrifugal motion on a primary axis and rotational motion on a secondary axis, the wall of a DAC mixing vessel essentially acts as a shearing blade. DAC is generally reproducible and has several advantages over traditional mixing methods (2,3). With no need for a central mixing component that is removed after processing, yield is improved and cumbersome cleaning procedures between batches are eliminated. Additionally, batches can be sealed, sterilized, and processed directly into their final container closure system (4). DAC excels in processing lab-scale volumes (< 1 g) (5) enabling high sample number, and low sample volume formulation screening studies (6–8). DAC utilization in the pharmaceutical space seems relatively novel still, but notable applications include siRNA-loaded liposomes (5), vesicular phospholipid gels (9–11), and fat emulsions (12).

Pirfenidone (Pf; 5-methyl-1-phenyl-2[1H]-pyridone; MW: 185.22 g/mol), which belongs to a class of medications called pyridones, is an orally active, synthetic small molecule that exhibits anti-fibrotic and anti-inflammatory properties and is approved for the treatment of idiopathic pulmonary fibrosis in Europe, Japan, and the USA (13). There has been a growing interest in broadening its treatment effects on dermal-related fibrotic diseases and other injuries (14–18). Pf has been shown to have anti-fibrotic effects in a variety of cells types, both mesenchymal and epithelial, where it is well tolerated and not cytotoxic at therapeutic doses (19,20). Our group and others have demonstrated that Pf inhibits human dermal fibroblasts trans-differentiation to myofibroblasts, decreases collagen deposition and fibrosis-related gene expression, and reduces activation of p38 mitogen-activated protein kinase in transforming growth factor-beta 1 (TGF-β1)-stimulated human dermal fibroblasts (21,22). Recently, we demonstrated that Pf can weaken the contractile machinery of activated dermal myofibroblasts (23). Using a deep partial-thickness burn model, we also observed that a Pf ointment formulation imparted a dose-dependent reduction inflammatory cytokines and alpha-smooth muscle actin (α-SMA) expression in mouse burn wounds (24).

We sought to formulate Pf in an alternative topical drug delivery vehicle to improve on the performance of our previous petrolatum-based ointment. We were interested in poloxamer 407 (P407) for its reported implications in burn injury and scarring. Biologically, poloxamers insert into cell membranes and can be both reparative and protective from insults (25–27). For formulation functionality, P407 is a non-ionic surfactant that can act as an emulsifier, a drug solubilizer, and a viscosity enhancer and has been widely used in various pharmaceutical vehicles (28). Specifically in the topical/transdermal space, P407 has been notably used in pluronic lecithin organogels (PLO) (29,30). Furthermore, we sought to emulsify P407 with an appropriate oil phase to address the prevalence of insolubilized Pf that we previously observed in our petrolatum-based ointment formulation.

In this work, we used DAC to develop and produce a poloxamer nanoemulsion gel (PNG) for topical delivery of Pf. Due to the gelling behavior of solubilized P407, processing requires high-shear methods (*e.g.,* high-pressure homogenizer) or temperature regulation to efficiently blend a P407 gel into a final formulation (29). To our knowledge, the utility of DAC to emulsify a P407 aqueous gel has not been demonstrated. We hypothesized that DAC could be used to emulsify a room-temperature P407 gel with Pf, a triacetin oil phase, a polysorbate 80 stabilizer, and a benzyl alcohol preservative. We established processing parameters to prepare ranging batch sizes (1.5–100 g) that are reproducible and with acceptable drug loading homogeneity. Compositional screens were performed to compare the permeation of Pf in *ex vivo* full thickness porcine skin. Furthermore, we demonstrated that the formulation maintained the *in vitro* dermal anti-fibrotic activity of Pf. Lastly, we characterized the compositional stability in regard to *in vitro* drug release, pH, viscosity, % drug content, and droplet size.

## MATERIALS AND METHODS

### Materials

P407, triacetin, propylene glycol, polyethylene glycol (PEG) 400, oleyl alcohol, octyldodecanol, medium chain triglyercides, isopropyl myristate, cocoyl caprylocaprate, decyl oleate, and pyrrolidone were kindly provided by BASF (Florham Park, NJ, USA). Pf (99%, HPLC) was purchased from AK Scientific (Union City, CA, USA). Benzyl alcohol, dimethyl sulfoxide, potassium phosphate monobasic (KH_2_PO_4_), mouse monoclonal antibody against GAPDH (1:500, clone 6C5), Triton X-100, and Tween 20 were purchased from MilliporeSigma (Burlington, MA, USA). HPLC-grade water, acetonitrile, and methanol were purchased from Fisher Scientific (Hampton, NH, USA). S600 oil was purchased from Canon Instrument Company (State College, PA, USA). Regenerated cellulose membranes (MWCO 12-14 kDa) were purchased from Repligen (Waltham, MA, USA). Porcine skins were commercially purchased from Midwest Research Swine (Gibbons, MN, USA). Stocks of adult normal human dermal fibroblast cells (NHDF) from two separate donors were purchased from PromoCell (Heidelberg, Germany). Ten percent fetal bovine serum was purchased from HyClone, GE Healthcare Life Sciences (Issaquah, WA, USA). Recombinant human TGF-β1 was purchased from R&D Systems (Minneapolis, MN, USA). Paraformaldehyde (4%) was purchased from Electron Microscopy Sciences (Hatfield, PA, USA). Mouse monoclonal antibody against α-SMA (clone 1A4) was purchased from Abcam (Cambridge, MA, USA). Gibco normal goat serum, Dulbecco’s modified Eagle’s medium, penicillin-streptomycin, Hoechst 33342, Pierce RIPA buffer, Pierce EDTA-free protease inhibitors mini tablets, Pierce BCA assay, ProLong Diamond mounting solution, Alexa Fluor 488-conjugated goat anti-mouse secondary antibody, and TRITC-conjugated phalloidin were purchased from Thermo Fisher Scientific (Waltham, MA, USA).

### Poloxamer Nanoemulsion Gel Preparation

A stock of aqueous 30 wt% (g/g) P407 was pre-prepared by Eurostar 60 overhead mixing (IKA, Staurfen, Germany) with ice water. The nanoemulsion gel was additionally composed of triacetin, polysorbate 80, and benzyl alcohol (Table I). To prepare a batch, all ingredients were dispensed individually using a Repeater E3x electronic positive displacement dispenser (Eppendorf, Hamburg, Germany) calibrated to each ingredient’s liquid density. Briefly, a 30 wt% Poloxamer gel, chilled on ice to liquefy, was dispensed into a mixing cup and allowed to gel at room temperature. All other ingredients were dispensed appropriately, including the Pf powder. A mixing cup was capped and mixed on a SpeedMixer™ 150.1 FVZ dual asymmetric centrifuge (DAC) (FlackTek Inc., Landrum, USA). Parameter optimization included varying mix speed (1500, 2500, and 3500 RPM) and time (1, 2, and 5 min). Due to a 1 min limit on the equipment, extended mix times are the result of multiple 1-min spins. Batches were reproduced to *n* = 3, except for 100 g batch sizes which was prepared *n* = 1 due to drug costs. Penetration enhancers (cocoyl caprylocaprate, dimethyl sulfoxide, decyl oleate, isopropyl myristate, octyl dodecanol, pyrrolidone, propylene glycol) were also evaluated at 5 wt% of the vehicle in replacement of a proportional amount of water.

**Table I:** Composition of Poloxamer-Based Nanoemulsion gel (PNG)

Component	Function	wt%
Water	Water phase	47.8%
Triacetin	Oil phase	27.6%
Poloxamer 407	Viscosity enhancer/emulsifier	13.8%
Polysorbate 80	Co-emulsifier	1.8%
Benzyl alcohol	Preservative	0.9%
Pirfenidone	API	8%

### uHPLC Quantification of Pirfenidone

Pf was quantified by uHPLC (Vanquish™ Flex Quarternary, Thermo Scientific, Karlruhe, Germany) equipped with an INTERSIL ODS2 (150 Å, 5 μm, 4.6 mm × 250 mm) C18 column. Mobile phase consisted with 65:35 ratio of 0.02 M, pH 2.5 KH_2_PO_4_ in water:acetonitrile in isocratic mode with a flow rate of 1 mL/min and injection volume of 20 μL. The temperature was maintained at 30°C by air circulation in the column compartment, and Pf was detected at a wavelength of 310 nm with a retention time of approximately 9 min. The concentrations of unknowns were determined by comparing against a standard curve ranging from 3.9 to 500 μg/mL (Supplemental 1, *R*^2^ = 1.000, limit of detection (LOD) = 1.146 μg/mL, limit of quantification (LOQ) = 3.472 μg/mL).

### Drug Uniformity

Drug loading of Pf was determined by sampling from three different areas of every batch produced. Samples were diluted 100× in a 1:1 mixture of methanol:water and stirred for 1 h. Samples were syringe-filtered with a 0.44-μm PTFE membrane and ran on an uHPLC.

### Droplet Sizing

Dynamic light scattering (DLS) was used to determine nanoemulsion droplet sizing on a Zetasizer Nano ZS (Malvern Panalytical, Almelo, Netherlands). Nanoemulsion was diluted 500× in 10 mM potassium chloride (KCl) with vortexing. No significant difference in size measurement was observed when diluted 10–1000x, or by resuspension method of vortexing or stir bar (data not shown).

### Rheometry

Rheological measurements for nanoemulsion samples were performed using a Haake MARS III (Thermo Scientific, Karlsruhe, Germany). A 20-mm diameter titanium parallel plate was used as a measuring geometry. All measurements were performed at 22°C, which was controlled by using a Haake 10 water bath (Thermo Scientific, Karlsruhe, Germany), and recorded 1 day after preparation, then every week (for the 100 g batch) over 1 month as part of the stability test. Briefly, 400 μL of nanoemulsion sample was dispensed onto the surface of the lower plate. Then, the upper plate was lowered to a gap of 1 mm. Excess sample was removed by scrapping the edges of the plate using a disposable spatula. Four sequential steps were performed for rheological characterization. Step 1: The samples were allowed to relax from stress accumulated from loading by performing an oscillation time sweep test for 10 min (at strain, *γ* = 0.1% and frequency, *ω* = 1 Hz). Step 2: An oscillation stress sweep test was done (*γ* = 0.001–50%, *ω* = 1 Hz) to determine the linear viscoelastic region (LVER) for step 3. Step 3: An oscillation frequency sweep test was performed (*γ* = 0.01%, *ω* = 1–100 Hz) to characterize the nanoemulsion formulation. Step 4: Finally, a steady state rotation step was performed (shear rate from 0.0002 1/s to 50 1/s) to obtain the relationship between viscosity and shear rate. We report the zero-shear viscosity obtained from step 4 as part of the 1-month stability test. Zero-shear viscosity is the maximum plateau value obtained at very low shear rates; this is the viscosity of the formulation at rest. Furthermore, Ostwald-de Waele model was used for fitting the plot between shear stress (*τ*) and shear rate ($\frac{\partial u}{\partial y}$); this model gives the value of consistency index (*K*) and flow behavior index (*n*).

1 $$\tau =K{\left(\frac{\boldsymbol{\partial u}}{\boldsymbol{\partial y}}\right)}^{\boldsymbol{n}}$$

S600 oil was used as a viscosity standard to verify the accuracy of rheometer (Supplemental 2). Briefly, 400 μL of S600 oil was dispensed on the lower parallel plate. Then the upper plate was lowered to a gap of 1 mm. The shear rate was varied from 0.1 to 1000 1/s, and shear stress was measured *via* rotation step (stage temperature used was 20°C). Based on the plot in Supplemental 2, shear rate *vs* shear stress plot resulted in a linear relationship with R2 value of 0.9989 and slope (dynamic viscosity) of 1.567 mPa-s (reported manufacturer value is 1.594 at 20°C).

### *In Vitro* Release Test

*In vitro* release tests (IVRT) were performed on a vertical diffusion cell (Vision® Microette™, Hanson, Chatsworth, CA, USA) with automated sampling. Diffusion cells had an effective diffusion area of 1.77 cm^2^ and a donor chamber height of 1 mm. Donor chamber was separated from receptor chamber with a regenerated cellulose membrane of MWCO 12–14 kD. Receptor chamber was 6.7 mL in volume and filled with 1× phosphate-buffered saline (PBS) at pH 7.4 and maintained at 32°C using a recirculating water bath. A total of 500 μL samples were taken at 1, 2, 3, 4, 6, 8, 12, 18, and 24 h which involved a 1 mL flush followed by a sampling event. Pf concentration in all samples was quantified by uHPLC.

### *Ex Vivo* Deposition in Porcine Skin

Deposition of Pf (8 wt%) from different compositions was screened on commercially sourced full-thickness porcine skin from Midwest Research Swine (Gibbon, MN, USA). Uncompromised skin visually free of redness or blemishes was collected at 3-mm thickness from the back of a single donor and shipped on ice by the supplier. Once received, skins were first frozen at -20°C before being die press cut at 3/4″ diameter to fit our custom vertical diffusion cell adapter and stored at -20°C until use. The adapter consists of a lip that applies downward pressure on the outside edges of the skin to limit formulation leakage. At the time of the experiment, frozen skins were inserted into the skin adapter with effective diffusion area of 1.77 cm^2^ and mounted on a diffusion cell that was set to 37°C. The receptor was filled with 1× PBS containing 0.05 wt% gentamycin to limit microbial growth. Skins were equilibrated to room temperature for 1 h before applying formulation to the donor chamber. PNG samples with chemical penetration enhancers at 5 wt% or without were separately tested and applied as a 1-mm-thick layer (approximately 200 mg) to assess the effect on Pf deposition in skin. The system was occluded and allowed to sit for 24 h, and then the skin and receptor fluid were collected. The surface of the skin was wiped with an alcohol wipe to remove residual formulation from the donor phase. Pf was extracted from intact skin by incubating in 10 mL of methanol for 24 h at 50°C in a sealed, 20 mL glass scintillation vial to minimize solvent evaporation. All skin extracts and receptor fluids were syringe-filtered with a 0.44 μm nylon membrane prior to quantification by uHPLC.

### *In Vitro* Biological Activity Assay in TGFβ-1 Human Dermal Fibroblasts

Normal human dermal fibroblasts (NHDF) were maintained in complete media (DMEM, 10% FBS, 1% penicillin-streptomycin), and experiments were performed using cells cultured within 8 passages. Cell assays were performed as previously described (21). For both immunocytochemistry and Western blot assays, cells were seeded in complete media and grown for at least 24 h before serum starvation in serum-free media (SFM) with 1% penicillin-streptomycin. After 24 h in SFM, cells were treated with 10 ng/ml TGF-β1 with or without Pf (0.5 mg/ml). Fresh Pf solution dissolved in 1× PBS was compared with Pf released from gel preparations into PBS. Pf extraction consisted of dialyzing vehicle or vehicle with drug in a regenerated cellulose dialysis tube (MWCO 12-14 kDa) and collecting the release fractions after incubation at 37°C for 24 h with rocking. The concentration of fresh and released Pf was verified by uHPLC before solutions were filter sterilized (0.22 μm).

### Immunocytochemistry and Confocal Imaging

NHDF were seeded at 2500 cells per well in 8-well chambered slides (Nunc Lab-Tek, Thermo Scientific). Serum-starved cells were treated with 10 ng/ml TGF-β1 and vehicle or gel-released Pf (0.5 mg/ml) and cultured for 4 days before fixation for 20 min in 4% paraformaldehyde. After 10 min permeabilization in 0.1% Triton X-100 and 1 h blocking with 10% normal goat serum, 0.1% Tween 20 in PBS, slides were incubated for 1.5 h at room temperature in primary antibody diluted in blocking solution (1:100, mouse monoclonal against α-SMA, clone 1A4). After three 5 min rinses in PBT (0.1% Tween 20 in PBS), slides were incubated for 1 h at room temperature with secondary antibody Alexa Fluor 488-conjugated goat anti-mouse (1:1000), TRITC-conjugated phalloidin (1:40), and Hoechst 33342 (1:2000). All dilutions were prepared using the blocking solution. Slides were mounted with ProLong Diamond mounting solution. Confocal images were acquired with a Plan-Apochromat 10×, 0.45 NA objective on a Zeiss 710 confocal microscope (Zeiss, Thornwood, NY). The same laser power, gain, and digital offset were used for all slides. Image montages were prepared in ImageJ (National Institutes of Health, Bethesda, MD).

### Western Blot Analysis

NHDF were seeded in complete media at 8 × 10^4^ cells per well in 6-well tissue culture plates and grown to 70% confluence before 24 h serum starvation and subsequent treatment. NHDF were directly lysed on the culture plate in Pierce RIPA buffer supplemented with Pierce EDTA-free protease inhibitors mini tablets. Plates were then scraped to collect whole cell lysates before samples were clarified by centrifugation at 14,000 RPM for 15 min at 4°C. Protein concentration was quantified using the Pierce BCA assay. Immune detection of proteins was performed using the Simple Wes capillary-based Western blot technology as described previously (31) (Protein Simple, San Jose, CA). Briefly, samples were adjusted to equivalent protein concentration using provided sample buffer; four-part protein sample was mixed with one part provided 5X master mix (Protein Simple) containing fluorescent molecular weight markers and DTT (40 mM). Samples were heated at 95°C for 5 min prior to loading into supplied microplate. Primary antibodies for α-SMA (1:20) and GAPDH (1:500) were diluted in provided antibody diluent and added to microplate along with provided HRP-conjugated secondary antibody, chemiluminescent substrate and wash buffer. The automated capillary system, Simple Wes, separated the proteins by electrophoresis, immobilized the proteins, and then performed immunoblotting in the same capillary. Protein quantification was performed using the Compass software for Simple Western (v3.0.9; Protein Simple).

### Formulation Stability

Formulation stability was monitored over the course of 1 month at normal (25°C/60% RH) and accelerated (40°C/75% RH) conditions as outlined by ICH Q1A(R2). Formulation pH, viscosity, drug content, and droplet size were measured at days 0, 7, 14, 21, and 28. The effect of storage on IVRT was assayed at 28 days. pH was determined by diluting the formulation 10× in filtered HPLC water and measuring with a SevenExcellence pH meter configured with an InLab Expert PRO-ISM pH electrode (Mettler Toledo, Columbus, OH, USA). All other characterization was performed as detailed previously.

### Statistical Analysis

All data analysis was done on GraphPad Prism 8 software. Statistical significance of differences in drug homogeneity was determined with a two-way ANOVA followed with *post hoc* testing at a confidence level of 0.05. Differences in Pf permeation for various penetration enhancers were evaluated by the Student’s *t* test with a confidence level of 0.05. Statistical significance for *in vitro* activity assay was determined by one-way ANOVA followed with Bonferroni post-test at a confidence level of 0.01. All results are reported as average ± standard deviation (stdev).

## RESULTS

### Processing Parameter Determination

We initially optimized DAC processing parameters using a small, 1.5 g batch size. The time and mixing speeds were the primary setting inputs for DAC, and we wanted to establish a working range that could effectively dissolve Pf and emulsify pre-solubilized P407 gel with additional excipients in a single step. We tested variations to mixing speed (1500, 2500, and 3500 RPM) and time (1, 2, and 5 min) using the drug homogeneity as the primary criterion of acceptability according to USP standards: < 6% relative standard deviation and no individual samples outside 10% of the label. Three individual batches were produced for each setting combination.

**Fig. 1:**
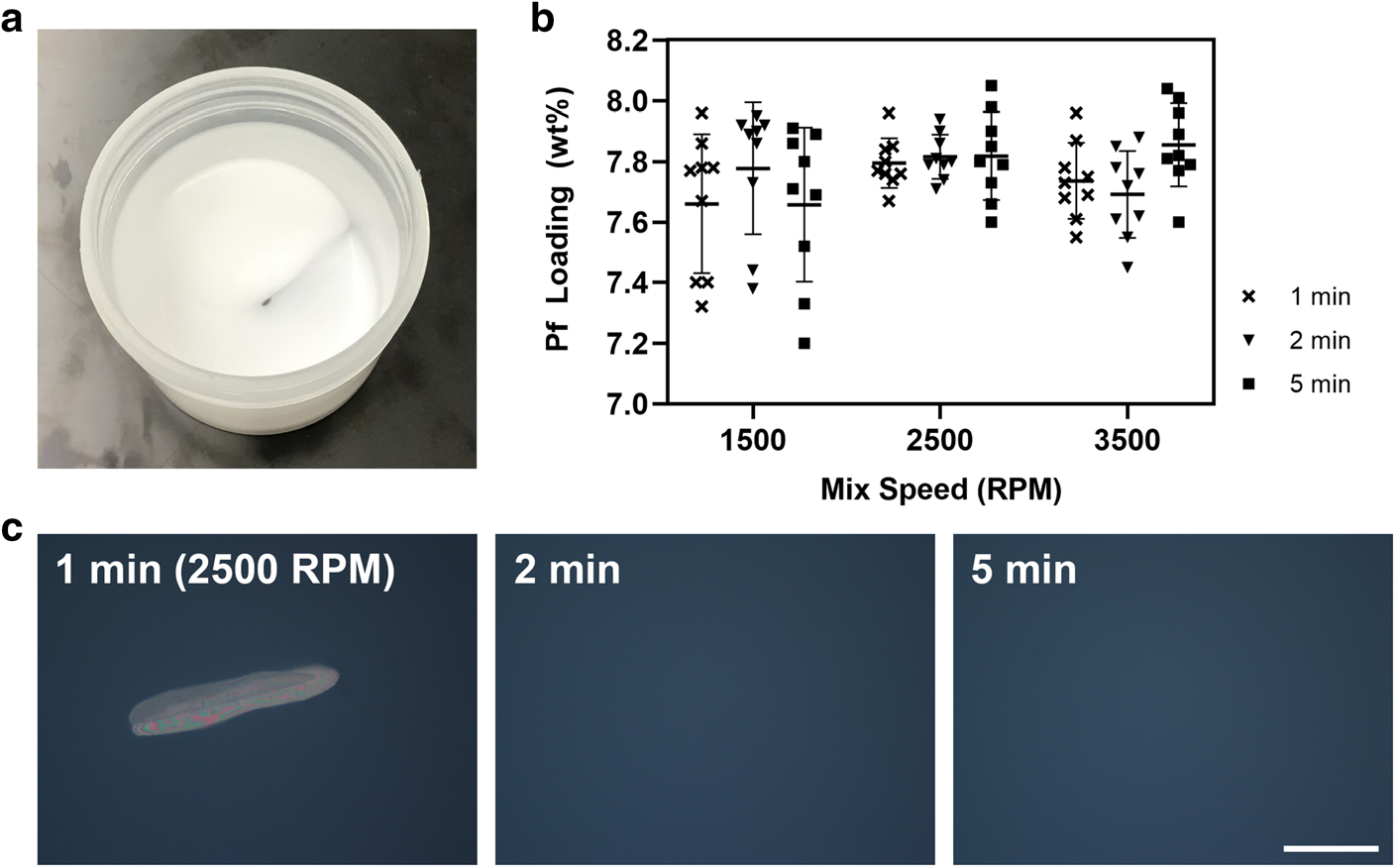
DAC parameter optimization for mix speed (1500, 2500, and 3500 RPM) and mix time (1, 2, and 5 min). **a** PNG is visually white, smooth, non-flowing, and free of bubbles. **b** Drug loading and homogeneity were measured and showed tight distribution of samples when processed at 2500+ RPM. **c** Representative cross-polarized microscopy images show presence of undissolved Pf when mixed for only 1 min, but not at 2+ min when processed at 2500 RPM. Scale bar is 100 μm. *n* = 3 batches, 3 samples per batch. Standard deviation bars = avg. ± stdev

Descriptively, the PNG produced by DAC was white in color, smooth, non-flowing, and bubble-free (Fig. 1a). After altering mixing speed and time for a 1.5 g batch size, a two-way analysis of variance (ANOVA) revealed that speed (*F*(2,72) = 2.997, *p* = 0.06) impacted drug homogeneity more than mix time (*F*(2,72) = 0.534, *p* = 0.60). By increasing mix speeds over 1500 RPM, there was a noticeable tightening distribution of samplings for drug loading (Fig. 1b). For example, comparing a 2 min mix at 1500 RPM to 2500 RPM resulted in improved drug loading uniformity with standard deviations of 2.79% and 0.96%, respectively. While a 1 min spin was enough to meet USP guidelines on drug homogeneity, immediate observation under cross-polarized microscopy showed the presence of insolubilized Pf (Fig. 1c). This was true for all mix speeds, but Pf was fully dissolved by increasing mix time to 2 min and over. For further experiments, we used a 2500 RPM mix speed for 2 min to minimize process time and avoid potential over-mixing.

### Batch Size Processing

A major advantage of DAC is the feasibility to produce small batch sizes that are difficult to process with traditional mix methods. Smaller batches can result in cost saving when scouting process settings and screening compositions. Using a 1.5 g batch size, we identified that a 2500 RPM mix for 2 min can simultaneously emulsify the vehicle and homogenously distribute Pf. Maintaining the same process settings, 1.5 g, 15 g, and 100 g batch sizes were produced and their drug homogeneity and rheological properties were compared for further characterization studies.

**Fig. 2:**
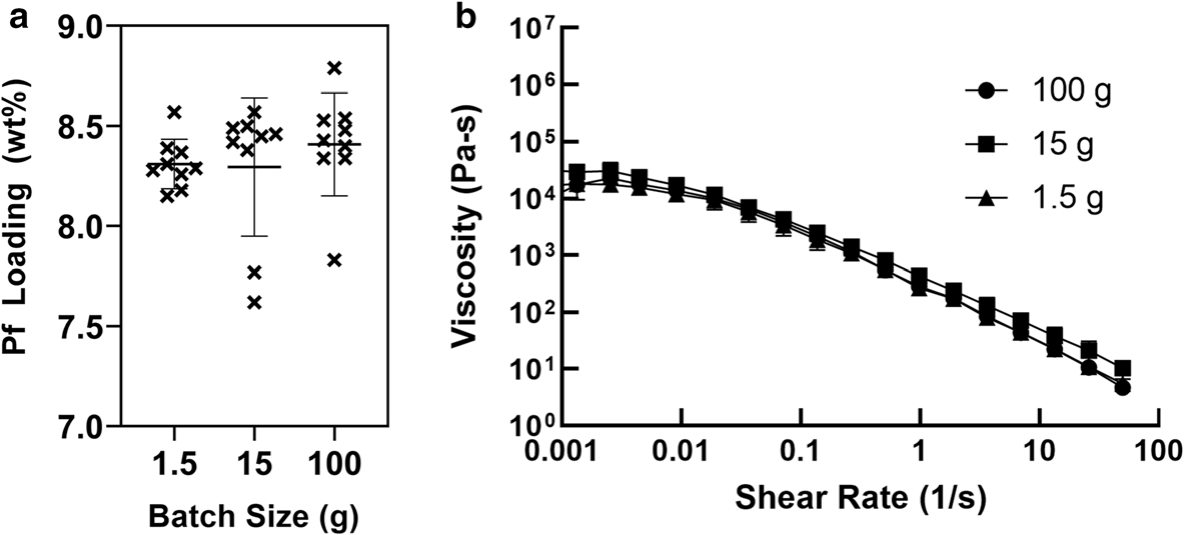
Batch size processing. With a constant processing setting of 2500 RPM for 2 min, 1.5, 15, and 100 g batch sizes were characterized to determine **a** drug loading and homogeneity and **b** viscosity. Standard deviation bars = avg. ± stdev

Compared with a 1.5 g batch size, 15 g and 100 g batches showed a broader distribution of drug loading measurements when sampled across different areas of the mixing cup (Fig. 2a). This indicated that larger batches may require either longer mix times and/or faster mix speeds in order to compensate for the larger volume of material that Pf must be dispersed into. In this preliminary scalability evaluation, we demonstrate the same setting can apply to batch sizes from 15 to 100 g with drug loadings of 8.30 ± 0.35 wt% (4.18% RSD) and 8.41 ± 0.26 wt% (3.05% RSD), passing USP guidelines on drug uniformity.

In addition to measuring drug loading and homogeneity, we added rheological characterization to compare PNGs produced at ranging batch sizes. The rheological evaluation showed that viscosities were comparable across the three batch sizes (Fig. 2b). Moreover, the dependence of viscosity with shear rate and its flow behavior was consistent with a non-Newtonian pseudoplastic material. This is typical for a concentrated emulsion formulation such as our PNG (32). Moreover, when shear stress *vs* shear rate plots were fitted with Ostwald-de Waele model (power law), the flow behavior index (*n*) gave a value of less than 1 for all batch sizes, which is indicative of a pseudoplastic type of fluid (Supplemental 3A). In addition, a horizontal-parallel behavior between storage modulus (G′) and loss modulus (G″) with G′ (elastic contribution) being larger in value than G″ (viscous contribution) (Supplemental 3B) indicated that the PNG had a gel-like consistency (*i.e.,* elastic-like behavior); this plateau region is indicative of an entangled polymer system. At this plateau region, the PNG reached stability at rest due to no flow and behaved like a solid substance. Moreover, tan delta values (the ratio between G′ and G″) at this region showed values less than 1 for all batch sizes (Supplemental 4). Hence, the formulation retains its structure at rest without sedimentation or separation of phases. This was true for a range of frequencies, but as the frequency was increased, both G′ and G″ increased, and G″ approached G′, indicating that the force applied is higher than the microstructure forces. This leads to the collapse of the microstructure and the material starts to flow. At high frequencies, the PNG started to exhibit fluid-like behavior conducive to application on surface. These observations were consistent across batch sizes.

### *Ex Vivo* Porcine Skin Deposition of Pf

**Fig. 3:**
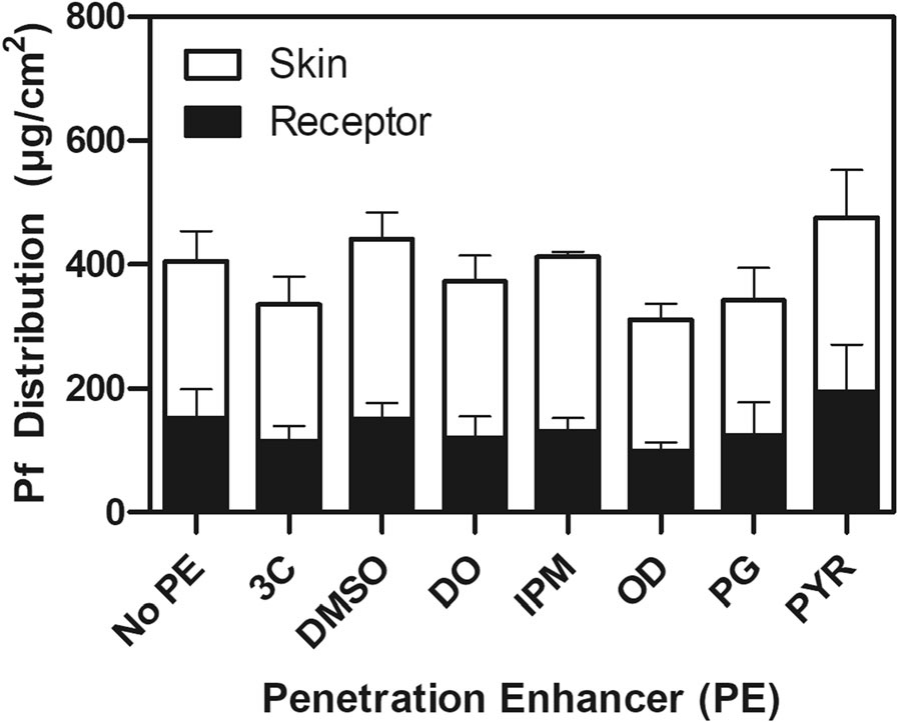
Effect of chemical penetration enhancers (PE) on Pf deposition in *ex vivo* porcine skin after 24 h. Cumulative Pf permeation was measured from skin extracts (white bar) and the receptor chamber (black bars). Cocoyl caprylocaprate (3C), dimethyl sulfoxide (DMSO), decyl oleate (DO), isopropyl myristate (IPM), octyldodecanol (OD), propylene glycol (PG), and pyrrolidone (PYR) were added at 5 wt% of the formulation. *n* = 3, single skin donor; standard deviation bars = avg. ± stdev

A panel of PNGs containing 8 wt% Pf and different chemical penetration enhancers was tested by applying a 1-mm-thick layer of PNG on a full thickness porcine skin (3 mmthick, 1.77 cm^2^ effective area) to assess differences in Pf deposition. Chemical penetration enhancers (PEs) were added at 5 wt%. This assay was used to measure Pf retention in full thickness skin as opposed to the more traditional *in vitro* permeation test (IVPT) in which pass through is only measured across thinly dermatomized skin. We found that after 24 h, the base composition resulted in a total of 404.90 ± 67.07 μg/cm^2^ of Pf permeated into the skin, 252.26 ± 48.51 μg/cm^2^ retained in the tissue, and 152.64 ± 46.32 μg/cm^2^ measured in the receptor (Fig. 3). Among the tested PEs, the highest permeation in the skin was associated with dimethyl sulfoxide (440.70 ± 50.95 μg/cm^2^) and pyrollidone (475.83 ± 107.99 μg/cm^2^), both aprotic solvents known to readily permeate through skin, but the increases were not statistically significant over baseline by Student’s *t* tests. Pf is a small molecule that has been previously demonstrated to permeate through skin with an optimized liposomal formulation resulting in ~ 500 μg/cm^2^ cumulative permeation at 24 h (15). Higher concentrations of excipients may be explored in the future to further enhance Pf delivery. We opted to continue characterization efforts with the baseline composition because the baseline was performed as well as formulations containing PEs in our initial screen.

### *In Vitro* Biological Activity of Gel-Released Pf

**Fig. 4:**
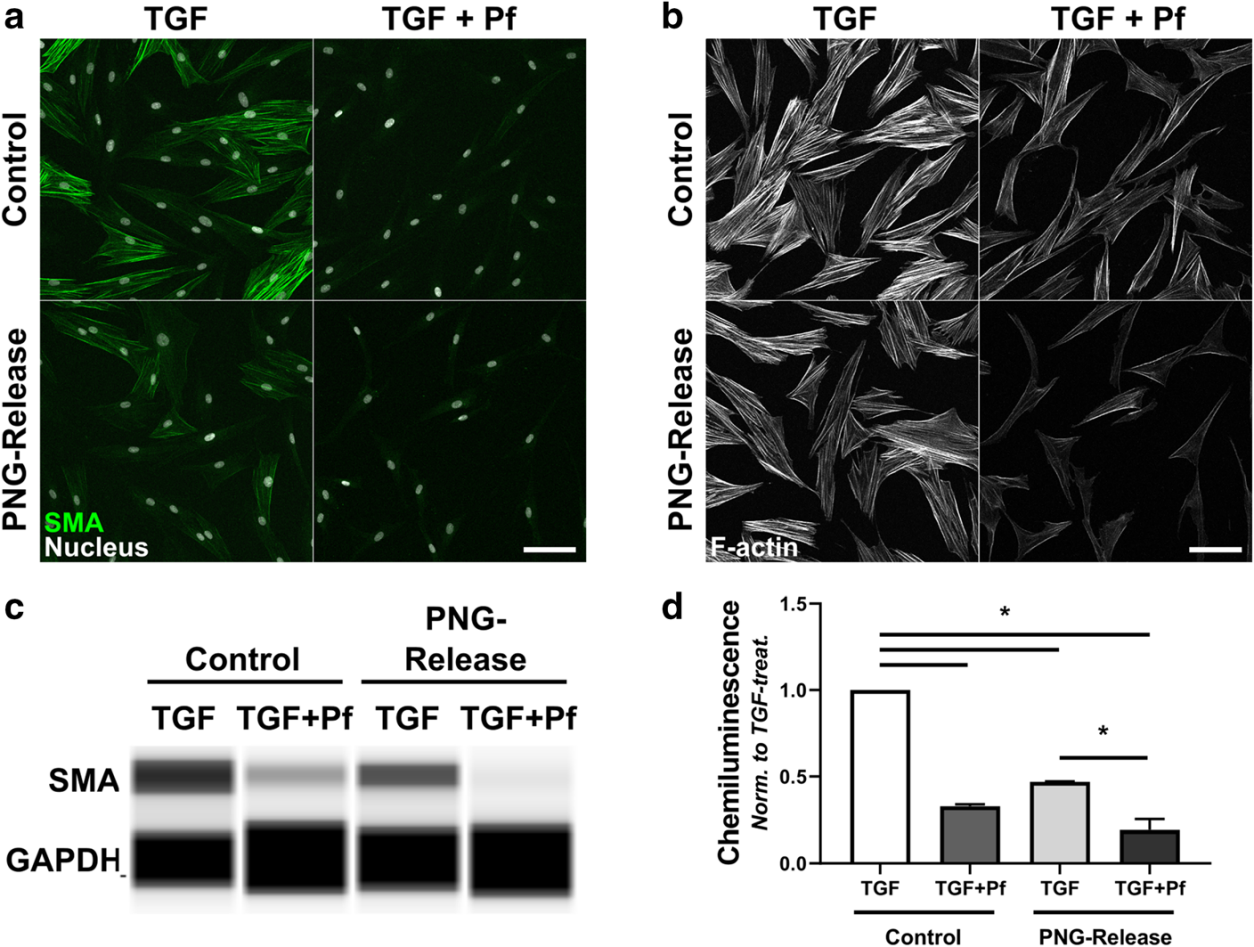
Pf retains biological activity in a PNG formulation. Fibroblasts were treated for 4 days with TGF-β1 alone (TGF, Control), TGF-β1 + Pf from powder in PBS (TGF + Pf, Control), TGF-β1 with vehicle release from blank PNG (TGF, PNG-Release), or TGF-β1 with Pf release from PNG (TGF + Pf, PNG-Release). **a** Fixed cells immunostained for α-SMA (green, SMA) and for nuclei with Hoechst (white, Nucleus). **b** Same treatments as in (**a**), F-actin stress fibers labeled with fluorescent phalloidin (F-actin). Scale bars are 100 μm. **c**, **d** Whole cell lysates were collected from fibroblasts treated as above and probed for α-SMA protein expression. **c** Pseudogel image of individual capillaries run on Simple Wes system probed for α-SMA and GAPDH. **d** Plots show avg. ± stdev from 2 donors, normalized to TGF-alone for each donor. α-SMA protein normalized to GAPDH probed in same capillary. *P < 0.01 with one-way ANOVA and Bonferonni post-test

Immunocytochemistry and protein expression analysis confirmed that Pf released from the PNG formulation retained its biological activity and inhibited myofibroblast differentiation (Fig. 4). Just as with the fresh Pf solution prepared from powder, the formulation-released Pf solution inhibited α-SMA expression and F-actin stress fiber formation (Fig. 4*a*, *b*). Although the TGF-β1 + vehicle treated samples showed lower α-SMA expression, there was a distinct further reduction of α-SMA expression with Pf released from the formulation. The protein expression analysis performed by Simple Wes capillary electrophoresis and immunodetection confirmed the immunocytochemistry results (Fig. 4*c*).

### Stability Assessment

**Fig. 5:**
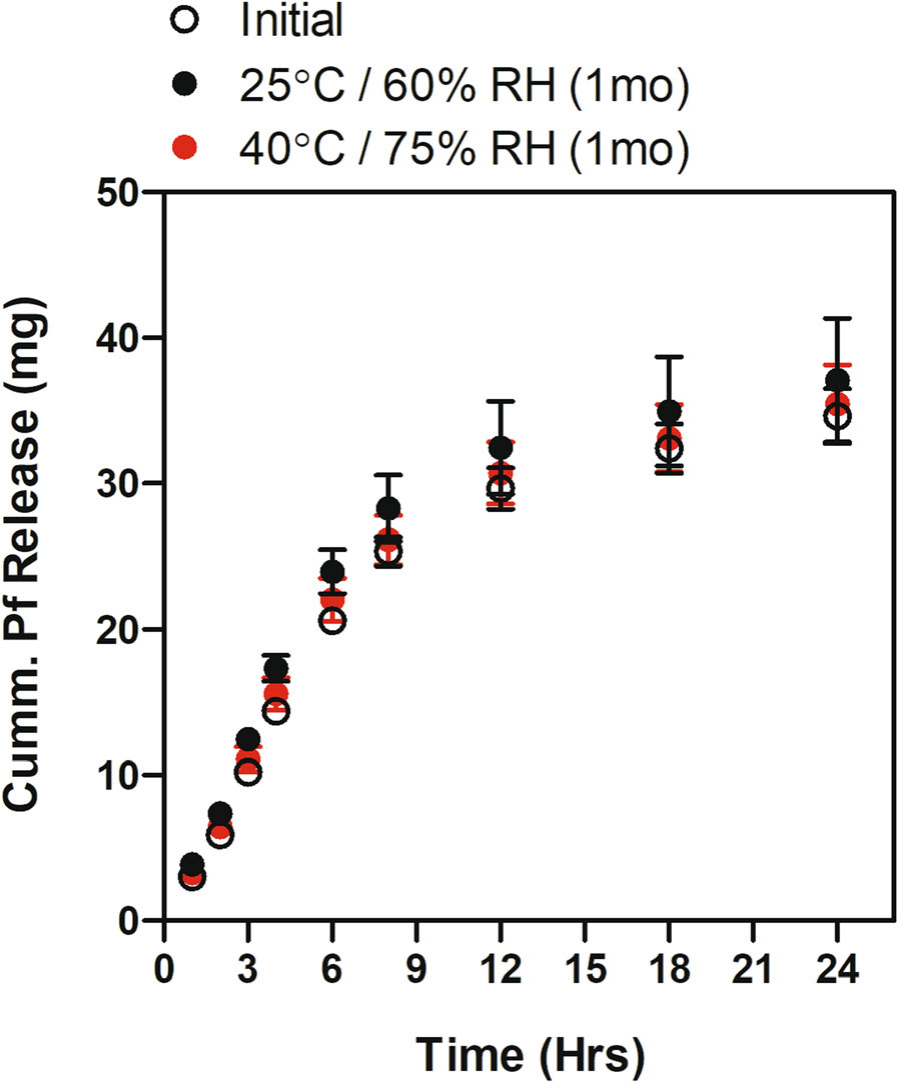
IVRT of Pirfenidone from PNGs. Release profiles were measured for freshly prepared formulation as well as formulations stored for 1 month at 25°C/60% RH and 40°C/75% RH. *n* = 3; standard deviation bars = avg. ± stdev

IVRT was performed to determine release profiles of Pf from PNGs and to assess the stability when stored at normal (25°C/60% RH) and accelerated (40°C/75% RH) conditions. Pf was released in a biphasic profile with a linear cumulative release out to 8 h, followed by a slower linear release out to 24 h (Fig. 5). Batches stored for 1 month at both conditions showed comparable release profiles to a freshly prepared batch. Notably, we observed that the PNG within the donor chamber becomes completely clear within 6 h (data not shown) which we suspect is due to the dissolution of the oil phase into the receptor phase as triacetin is slightly water soluble (60 g/L)(33).

**Fig. 6:**
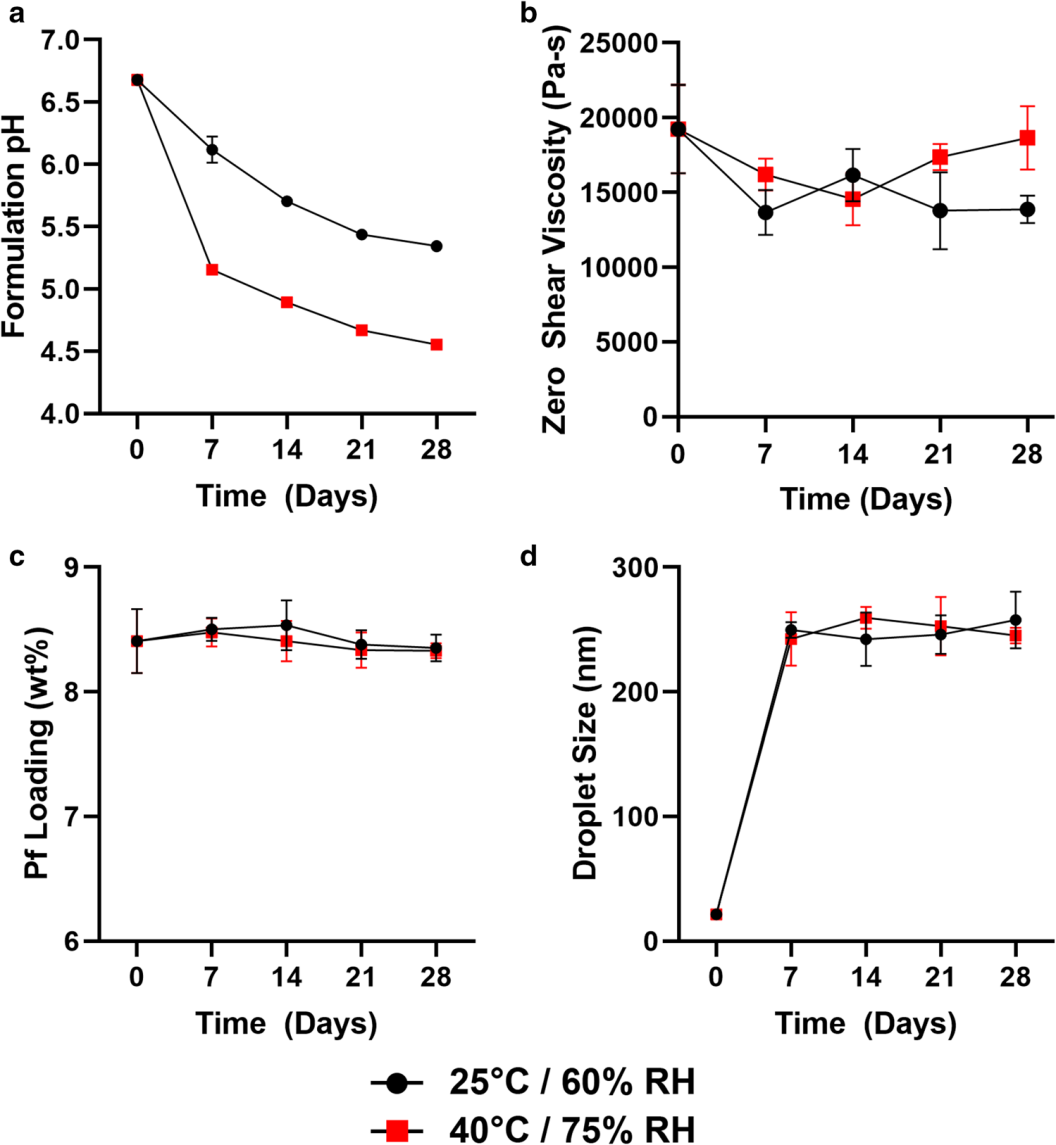
One-month stability assessment of PNG formulations. Stability was determined by **a** formulation pH, **b** viscosity at rest (zero-shear), **c** % drug content of day 0 loading, and **d** droplet size. *N* = 3; standard deviation bars = avg. ± stdev

The 1 month stability of our PNG composition was also assessed by pH, viscosity, drug content, and droplet size when stored at normal (25°C/60% RH) and accelerated (40°C/75% RH) conditions (Fig. 6a–d). Initial pH of the PNG was measured at 6.7 with marked decreases within 1 week, showing continued pH drop to 5.5 (25°C/60% RH) and 4.5 (40°C/75% RH) after 1 month (Fig. 6a). Observations of pH drop initially correlated with decreased viscosity over time; zero-shear viscosity decreased from 19.2 ± 3.0 MPa-s to 13.9 ± 1.5 MPa-s after 1 month storage at 25°C/60% RH, whereas it decreased from 19.2 ± 3.0 MPa-s to 14.5 ± 1.8 MPa-s after 2 weeks of storage at 40°C/75% RH but recovered at 1 month (Fig. 6b). Droplet sizing was initially ~20 nm, and for both storage conditions, significantly increased to ~ 250 nm within 1 week (Fig. 6d). Droplet size appeared to stabilize after 1 week and was relatively constant out to 1 month.

While these trends indicate dynamic changes to the formulation that can potentially impact biological efficacy, we observed no effect on drug content and biological activity of Pf. After 28 days, compared with a day 0 baseline, drug loading measurements showed 98.5 ± 1.3% (25°C/60% RH) and 98.2 ± 0.8% (40°C/75% RH) of Pf remained compared with a day 0 baseline (Fig. 6c). When Pf released from a 1 month stored PNG was added to TGF-β1-stimulated fibroblasts, α-SMA expression was visibly reduced and biological activity was maintained (Supplemental 5).

## DISCUSSION

In this work, we utilized DAC to emulsify a PNG composed of an aqueous phase containing P407 and an oil phase with high loading capacity for Pf as a topical vehicle for Pf. Triacetin was selected as an oil phase from a solubility screen in which Pf saturated at 7 w/v% (Supplemental 6). While propylene glycol and polyethylene glycol 400 have higher saturation solubilities compared with triacetin, these solvents could not be emulsified with a P407 gel because of their hydrophilicity. We observed that a P407 concentration > 10 wt% resulted in a gel-like consistency, and we included P407 at a final concentration of 13.8 wt%—lower than the 15 wt% maximum approve concentration in the FDA inactive ingredient database. Polysorbate 80 and benzyl alcohol are widely used pharmaceutical excipients and were used as a stabilizer and preservative, respectively. To emulsify this composition (Table I), we observed that DAC is very efficient and did not require long mix times.

To ensure that the processing and materials used in the PNG formulation did not diminish its established biological effect of Pf, we assayed the ability of the drug to inhibit human dermal myofibroblast differentiation. The persistence of highly contractile dermal myofibroblasts is a key feature of pathological scarring, such as hypertrophic scarring after burn injury (34). During wound healing, fibroblasts transform into contractile myofibroblasts upon exposure to TGF-β1 released by activated platelets and immune cells in the wound bed (35). These myofibroblasts express the alpha-smooth muscle isoform of actin (α-SMA) in contractile F-actin stress fibers that allow for greater force production to rearrange and contract surrounding extracellular matrix. Pf has been shown to inhibit the development of the myofibroblast phenotype in TGF-β1-treated normal human dermal fibroblasts *in vitro* by reducing the development of F-actin stress fibers and α-SMA expression (21). We did not observe noticeable loss of either drug content or drug activity with 1 month of storage. This is consistent with reports that Pf is a stable active pharmaceutical ingredient with minimal breakdown observed during forced degradation conditions including extreme pH, oxidation, light, and heat (36).

Additionally, we observed significant vehicle effects inhibiting human dermal myofibroblast differentiation. In support of these findings, poloxamers have been demonstrated to be protective in various injuries and pathologies including brain injury (25), rotator cuff injury (37), and cardiovascular disease (27), and can reduce fibrotic adhesions after surgery (38,39). Most relevant to our goal of a topical burn treatment, poloxamers have been reported to repair cells after heat shock insult (39) and may be useful in treating burn injury (40). Studies demonstrating the protective and reparative effects of poloxamers have primarily been with poloxamer 188 (27,41), but the broader poloxamer family has been demonstrated to have membrane repairing properties, including P407 used in our formulation (26). It is proposed that poloxamers function through direct insertion into phospholipid bilayer to either reseal damaged cell membranes or protect cells from damage by increasing membrane rigidity (26). Reduced membrane fluidity is likely to have significant consequences to cell signaling occurring at the plasma membrane with ultimate consequences to cytoskeletal structure and function (42,43). This increase in membrane rigidity may partly explain the vehicle effects seen with TGF-β1-treated dermal fibroblasts. In addition, poloxamers have been shown to negatively affect cell adhesion to a collagen substrate and cell aggregation (44,45). Myofibroblast formation requires the formation of large super mature focal adhesions to an extracellular matrix (46) and thus, may be affected by inhibition of this process by poloxamer. The mechanism of vehicle effects reducing α-SMA expression in human dermal myofibroblasts was not directly interrogated in this study but would be an interesting subject for future investigation.

While drug activity was maintained, we observed negative trends in the stability of the PNG composition. We observed a large increase in droplet size within 1 week. Notably, when droplet size was measured by DLS, we consistently observed a peak size shift of shrinking diameter over time (data not shown), so we are reporting the dominant peak measured immediately after the PNG was diluted. We suspect small droplets produced initially under high shear forces quickly equilibrated with rest to a more stable droplet diameter. Fine emulsions have been demonstrated to have higher viscosities than coarse emulsions (32), and the measured increase in droplet size may partially contribute to the decrease in viscosity after 1 week.

Furthermore, with measured decreases in viscosity as well as pH of our current PNG composition (Fig. 6a–b), we hypothesize that poloxamer degradation is contributing to the changes. Poloxamers are A-B-A block copolymers consisting of ethylene oxide and propylene oxide and have been demonstrated to undergo oxidative degradation with byproducts of acetic acid and formic acid that correspond to drops in pH (47). As poloxamer is the gelling agent used in the PNG, poloxamer degradation would also be consistent with the decreases in viscosity, but additional tests must be run to identify degradation products and acid species. From 1 month stored samples, it is notable that changes in pH or viscosity did not affect the release profile of Pf which we believe is driven by passive diffusion. In order to address the observed instability, studies with antioxidants would directly assess whether poloxamer degradation is contributing to the change in pH and viscosity. We incorporated benzyl alcohol as a preservative/antioxidant in the formulation, but alternative antioxidants such as butylated hydroxytoluene (BHT) have been demonstrated to mitigate the degradation of poloxamer and could be an approach to improve formulation stability (47).

Alternatively, triacetin is known to have acid degradation byproducts, namely acetic acid (48). According to the Organization for Economic Co-operation and Development (OECD) assessment of triacetin, abiotic hydrolysis is observed in water with a half-life of 60.4 days at pH 7, 25°C, and is also susceptible to breakdown by biological enzymes (*e.g.,* esterases) (33). However, limited hydrolysis is observed at lower pH. With pH adjustment and inclusion of buffering agents to match the acidic pH of skin (49), excipient degradation may be mitigated in future studies.

The relevance and utility of DAC as a pharmaceutical mixing method is dependent on its ability to scale-up. As we have demonstrated in this work, DAC can efficiently emulsify and homogenously distribute drug for batch sizes ranging from 1.5 to 100 g, the maximum limit of our current model. Output can potentially be scaled up to 5 kg using existing off-the-shelf DAC units, but a limitation of this mixing method is that it is currently capped to small-scale manufacturing efforts. This maximum batch size simply does not compete with traditional mixing methods that have capacities upwards of 10,000 kg. Production scale-up is a non-unique challenge that must be regularly addressed in translating lab-scale formulations to pilot scale and eventually commercial scale manufacturing (50,51). If DAC is used for compositional screening and lab-scale development of novel formulations with a goal of commercial scale, significant efforts must go into identification of an alternate mixing method which includes process development and validation (52,53).

Although there are limitations on batch capacity of commercial scale, DAC-based formulation development may still translate well for specialized markets that utilize distributed manufacturing networks (54–56). For example, compounding pharmacies have taken on specialized services and treatments with local, small-scale production that centrally manufactured products cannot provide (57,58). As personalized medicine demands more custom therapies, there have been notable examples of on-site manufacturing of drug products including tablets and implantable systems (59). With its many advantages previously discussed, DAC could prove valuable for rapid on-site production of not only PNGs, but a wide variety of pharmaceutical formulations.

## CONCLUSIONS

We demonstrated that a short, single step mix time can efficiently emulsify a poloxamer-based gel with a triacetin oil phase and other excipients. Pf powder was simultaneously distributed homogeneously and solubilized through the vehicle during the emulsification process. Stepwise batch scale-up from 1.5 to 100 g showed comparable rheological properties although further process optimization may be undergone to improve drug homogeneity in larger batches. The composition or process method did not inhibit the *in vitro* biological activity of Pf. Although less effective compared with PNG-Pf, vehicle controls showed effects in reducing α-SMA expression and myofibroblast development. However, the current composition requires selection of alternative antioxidants and/or pH modifier excipients to mitigate stability issues observed with drops in pH and viscosity when stored over time. In summary, these findings show that DAC is a rapid developmental tool for formulations and should be considered for broader formulation applications in the future.

## Electronic Supplementary Material

ESM 1 (DOCX 592 kb)
