# Supplementary material for: Dual Asymmetric Centrifugation Efficiently Produces a Poloxamer-Based Nanoemulsion Gel for Topical Delivery of Pirfenidone
Source: AAPS PharmSciTech. 2020 Oct 2;21(7):265. doi: 10.1208/s12249-020-01798-7 (PMC7529632; doi:10.1208/s12249-020-01798-7)
Supplement: Supplementary file 1 — (DOCX 592 kb) [file 12249_2020_1798_MOESM1_ESM.docx]

Supplemental Figures


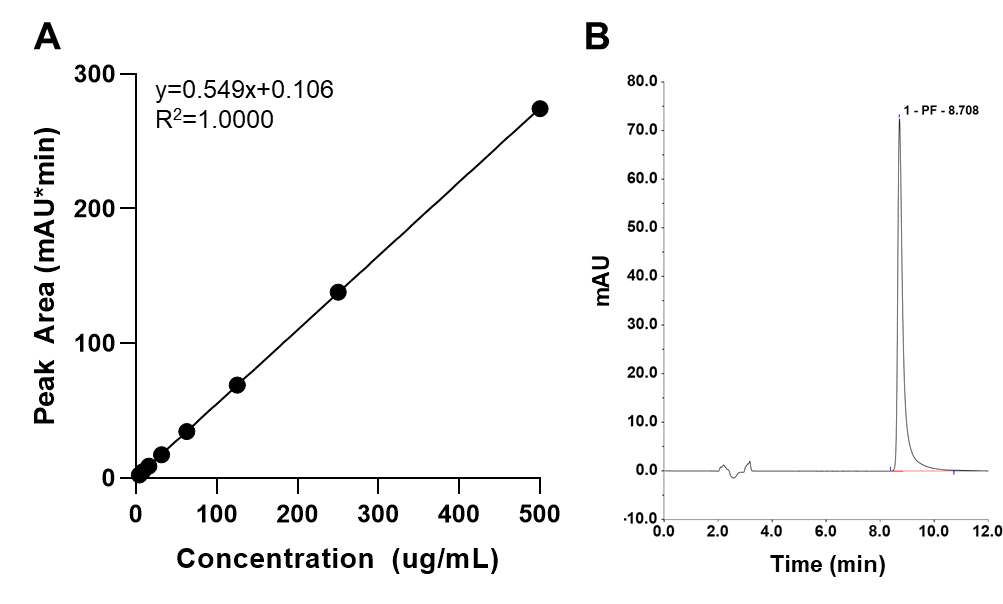
Supplemental 1. (A) Callibration curve for Pf quantification by HPLC and (B) representative chromotagraph of Pf.

Supplemental 2. S600 Oil viscosity standard for rheometer calibration.


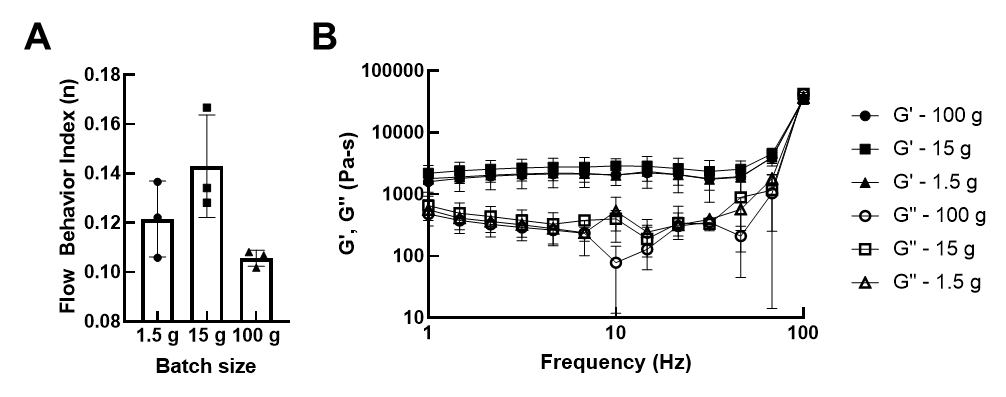


Supplemental 3. Poloxamer Nanoemulsion Batch-size scaleup. With a constant processing setting of 2500RPM for 2 minutes, 1.5, 15, and 100g batch sizes were characterized to determine (A) flow behavior index (n), and (B) storage and loss modulus vs frequency.

|  | 1.5 g Batch | 15 g Batch | 100 g Batch |
| --- | --- | --- | --- |
| Tan Delta | 0.06 ± 0.09 | 0.04 ±0.04 | 0.03 ±0.05 |

Supplemental 4. Tan delta values [ratio of storage modulus (G’) and loss modulus (G”)]. Values less than 1 indicates elastic-dominant (solid-like) behavior and values greater than 1 indicates viscous-dominant (liquid-like) behavior.

*
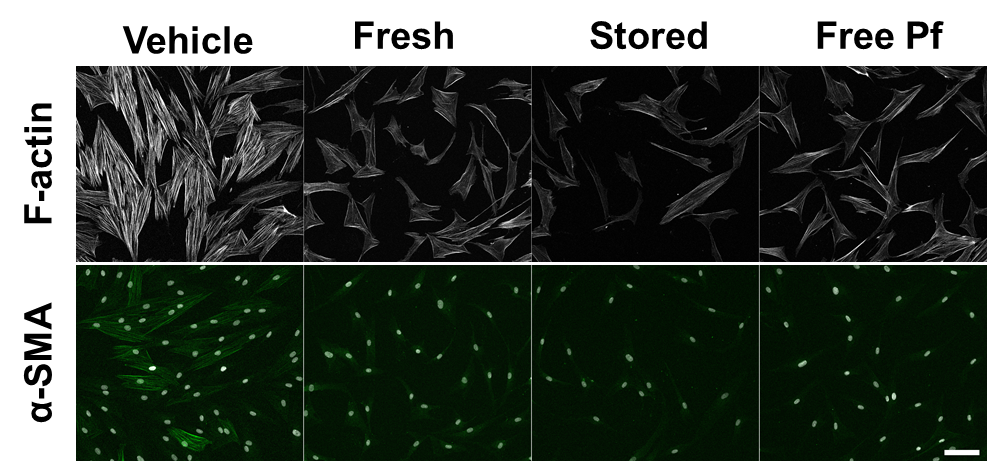
*

Supplemental 5. Biological activity was maintained in formulated Pf under fresh and storage conditions. Fibroblasts were serum starved and treated with 10 ng/mL TGF-β1 with release from blank gel (Vehicle) or 0.5 mg/mL Pf either prepared from powder (Free Pf), released from PNG formulation prepared fresh (Fresh), or from PNG stored at 25°C/60% RH for 1 month (stored). F-actin shown with fluorescent-labeled phalloidin staining. α-SMA (green) detected with immunocytochemistry and shown with nuclear counterstain (Hoeschst, white). Scale bar is 100 microns and applies to both image panels.

Supplemental 6. Pf saturated solubility in various hydrophilic and hydrophobic excipients.
